# Data sharing as social dilemma: Influence of the researcher’s personality

**DOI:** 10.1371/journal.pone.0183216

**Published:** 2017-08-17

**Authors:** Stephanie B. Linek, Benedikt Fecher, Sascha Friesike, Marcel Hebing

**Affiliations:** 1 ZBW—Leibniz Information Centre for Economics, Kiel, Germany; 2 German Institute for Economic Research, Berlin, Germany; 3 Alexander von Humboldt Institute for Internet and Society, Berlin, Germany; 4 VU University, Amsterdam, Netherlands; Institut Català de Paleoecologia Humana i Evolució Social (IPHES), SPAIN

## Abstract

It is widely acknowledged that data sharing has great potential for scientific progress. However, so far making data available has little impact on a researcher’s reputation. Thus, data sharing can be conceptualized as a social dilemma. In the presented study we investigated the influence of the researcher's personality within the social dilemma of data sharing. The theoretical background was the appropriateness framework. We conducted a survey among 1564 researchers about data sharing, which also included standardized questions on selected personality factors, namely the so-called Big Five, Machiavellianism and social desirability. Using regression analysis, we investigated how these personality domains relate to four groups of dependent variables: attitudes towards data sharing, the importance of factors that might foster or hinder data sharing, the willingness to share data, and actual data sharing. Our analyses showed the predictive value of personality for all four groups of dependent variables. However, there was not a global consistent pattern of influence, but rather different compositions of effects. Our results indicate that the implications of data sharing are dependent on age, gender, and personality. In order to foster data sharing, it seems advantageous to provide more personal incentives and to address the researchers’ individual responsibility.

## Introduction

At the present there is a lively discussion about open science and open data in the scientific community. Data sharing contributes to the quality and quantity of research. It enables data-driven replication studies and allows to pursue new research questions on the basis of secondary data [1,2]. However, even though the potential of openly available data is widely acknowledged, actual data sharing is rather limited [3–5]. For example: In a study among environmental scientists, Tenopir and colleagues [5] found that less than 6% of the surveyed researchers make all of their data available. Andreoli-Versbach and Mueller-Langer [4] found that that 81% of empirical economists do not voluntarily share their data. In a study among researchers from different disciplines, Fecher and colleagues [2] found that only 13% had actually made their own data publicly available in the past. Related prior research showed that most factors related to data withholding were associated with strategical publication considerations [1,2]. In addition, researchers partly did not make their data electronically available to others due to insufficient time and lack of funding [5]. In summary, there is convincing evidence that making data available for reuse is not considered a worthwhile practice among researchers. Instead, article publications are still the dominant currency in academia and the main vehicle for reputation gain.

Accordingly, academia can be described as a reputation economy, a system in which information and knowledge exchange is driven by a desire to accumulate reputation. However, so far making data available has little impact on a researcher's reputation. That is despite a little known second order effect: it has been shown that data publications increase the citation rates of the original published articles that describe the data collection [6]. Thus, Fecher and colleagues [1,2] argue that data sharing is a social dilemma: For the individual researcher there is a higher payoff to not investing time and effort into data sharing, but if everyone follows this selfish strategy, the scientific community will not benefit from open access to research data. In line with other authors, Fecher and colleagues [2] come to the conclusion that the transaction costs of making data reusable need to decrease and data sharing itself should receive more formal recognition, for instance via data citations, data awards, and additional funding [7,5,8].

While the literature mentioned above focuses on data sharing practices and related factors in a general system-related sense, the following paper explores the connection between data sharing and the researcher’s personality. We follow the “data sharing as social dilemma” argument and focus on the subjective perspective of the researchers and related interindividual differences within the current academic system. In contrast to prior work on data sharing behavior this contribution is less about the collective behavior in the scientific community or about interactions and cooperation between the researchers. Rather we focus on the subjective perspective of the researcher and how individual differences influence the subjective view (e.g., the attitudes towards data sharing and the perception of the situational factors) and the subjective decisions (e.g., the willingness to share and the actual data sharing).

Based on the appropriateness framework for decision making in social dilemmas [9] we argue that individual decisions about data sharing are based on the subjective perception of the data sharing situation in academia, the researcher’s identity, and the application of individual rules and heuristics (details on the appropriateness framework follow in the theoretical section). Accordingly, the overall aim of this contribution is to investigate the potential influence of the researcher’s personality on his/her data sharing behavior within an academic system that can be characterized as a reputation economy [2].

## Theoretical background

In this section we describe the theoretical background of our work. First, we outline data sharing as social dilemma within the appropriateness framework of decision making by Weber, Kopelman, and Messick [9]. In a second subchapter, we provide a short overview of selected personality factors and present empirical findings on their relation to workplace behavior and cooperation in social dilemmas.

### Data sharing as social dilemma and the logic of appropriateness

Social dilemmas are situations that fulfil two criteria: (a) they involve selfish, non-cooperative behavior that provides a higher payoff for the individual and (b) if everyone chooses the non-cooperative strategy all people received lower payoffs than if everybody cooperated [10]. In other words: “social dilemmas are situations in which individual rationality leads to collective irrationality” (p. 183 [11]). Other authors stress the temporal dimension and define social dilemmas as “situations in which short-term self-interest is at odds with long-term collective interests” (p. 125 [12]). This very broad definition includes social traps and real world problems like air pollution. In line with the characteristics of social dilemmas, in the current data sharing situation the individual researcher receives a higher payoff for non-cooperation (since data sharing has no impact on reputation but it is time-consuming) even though data sharing would be in the long-term interest of the research community.

Research on social dilemmas has a long tradition in psychology and several theoretical frameworks exist [12]. Many of the theoretical frameworks applied to social dilemmas are based on rational choice models and game theory [13]. Newer versions like the evolutionary game theory [14,15] comprise essential improvements and enhancements that enable the analysis of strategic choices, e.g., cooperation behavior in complex systems [16,17] and how reputation promotes the evolution of cooperation [18,19]. Evolutionary game theory is also capable to explain group interactions [15] and counterintuitive collective phenomena [14]. By analyzing the (repetitive) interactions between groups and individuals, evolutionary game theory addresses not only the temporal dimension but also the social nature of social dilemma.

In contrast to rational choice models, other approaches on social dilemmas like the appropriateness framework [9], stress not only the social nature of the dilemmas but also the importance of individual factors and the subjective view of the individual for cooperative behavior. The appropriateness framework is not suggested as a replacement for rational choice models but rather both approaches are designed for different application scenarios. Rational, economic choice models are very useful for situations without an obvious social context (fewer social features, large social distance between the involved persons, low level of communication and interaction) and for the explanation of collective phenomena. On the contrary, the appropriateness framework is more suitable when the situation is evidently social (e.g., social features and social norms are salient or communication is possible) and when the personal view might differ due to individual differences. As described in the introduction our contribution is less about interactions and collective phenomena and more about the subjective view and personal decisions of researchers. We therefore turn to the appropriateness framework to serve as the theoretical backbone of our investigation.

In principle, the logic of appropriateness [20] assumes that decisions are made on the basis of the question “what does a person like me do in a situation like this”([20] cited on p. 28 in [9]). This question includes three elements: the recognition of the situation, the person’s identity, and the application of rules. It is important to note that the individual perception of the situation is subjective, i.e., influenced by the individual characteristics of the decision maker. Additionally, the subjective classification of the situation is influenced by the normative context and the perceived social norms (what do others do). Within the appropriateness framework, identity is conceptualized as “an umbrella concept that includes all idiosyncratic factors that individuals bring with them into a social situation” (p. 283 [8]). The complex interplay between identity and situational features build up the person’s perception and categorization of the situation. This subjective definition of the situation is essential with respect to the behavioral choices (e.g., group task or individual task; competitive or cooperative situation). Situational features and behavioral options are subjectively perceived through the lens of identity. That means the subjective importance of specific conditions of a situation (incentives, barriers, punishments etc.) depends on the person’s identity and personal values.

Research on individual differences in social dilemmas supports the assumptions of the appropriateness framework. Numerous studies found individual differences in cooperative behavior in social dilemmas [9]. However, the findings on the concrete individual factors are somewhat inconsistent (see subsequent subchapters on personality domains), which underlines the complex interplay between situational features and the characteristics of the person’s identity.

Applied to data sharing in academia the appropriateness framework implies that the researcher’s identity influences not only actual data sharing behavior, but also how the researchers perceive the academic system and its possibilities. While actual data sharing behavior might be more influenced by constraints of the academic system, general attitudes towards data sharing and the willingness to share data should be more strongly influenced by the identity of the researcher and his/her perception of the relevant social norms. Similarly, the researcher’s subjective perception of the conditions of data sharing should be related with the researcher’s identity. Accordingly, the importance of enablers of and barriers to data sharing (like publication considerations and incentives for data sharing) also depends on the subjective view of the individual researcher.

Overall, besides the external systemic circumstances of data sharing, the researcher’s identity is a key factor in the social dilemma. Since identity is understood as an umbrella concept (that is hard to measure in its full broadness), we concentrated on some selected basic aspects, namely personality domains.

### Big Five, Machiavellianism and social desirability

Data sharing–or at least the decision to share data or not–is part of the scientific working behavior. In scientific literature, there is broad evidence for the connection between personality and working behavior in general [21–24] and counterproductive workplace behavior in particular [25–27]. There are also several findings on the influence of personality on knowledge sharing [28–30]. Since knowledge sharing implies conflicts of personal interest [29,31], knowledge sharing is very similar to the situation of data sharing.

Analogously, the personality of the researcher might influence data sharing within the current academic system that is driven by reputation gain. To our knowledge, so far there is no systematic empirical research on the connection between personality and data sharing behavior. The study presented in this paper aims at first insights in the complex interplay between the researcher’s identity and data sharing. Since identity is a very broad concept, we had to select some core characteristics. We focused on basic personality dimensions, on selected specific personality facets, and on basic sociodemographic variables.

Popular psychological approaches on basic personality dimensions are for example the so-called Big Five model [32,33] and the HEXACO model [34,35], which adds a sixth dimension (Honesty-Humility) to the Big Five. The Honesty-Humility dimension is partly similar with Machiavellianism, a personality facet we specifically wanted to assess. Thus (in order to avoid redundancy), we used the so-called Big Five as a basic concept for personality. Additionally, we included two specific personality facets that seemed to be of potential importance for the delicate topic of data sharing, namely Machiavellianism and social desirability. In addition, we included age and gender to control for basic sociodemographic variables. In the following we briefly describe the selected personality domains and related prior findings.

#### Big Five

The so-called Big Five model assumes five rather abstract factors as the basis for enduring individual differences in patterns of behavior, and experiences [32,33,36]. The five factors are extraversion (tendency to be communicative, gregarious, and outgoing), neuroticism (tendency to be nervous, sensitive, and emotionally unstable), openness (tendency to be imaginative, curious, and inventive), agreeableness (tendency to be friendly, compassionate, and cooperative), and conscientiousness (tendency to be organized, self-disciplined, and efficient).

Existing research on personality and general workplace behavior showed several relations of the Big Five with career success [23] and knowledge sharing [29,37,38]. For example, Matzler et al. [29] found positive relations between the willingness to share knowledge and the personality factors agreeableness, conscientiousness and openness.

The findings on the influence of personality factors in social dilemmas were not consistent. For example, Koole, Jager, van den Berg and Vlek [39] found in a resource dilemma more cooperation for low extraversion and high agreeableness. However, in longitudinal study of Volk, Thöni and Ruigrok [40] with a public goods game more cooperation was only associated with high agreeableness, but not with low extraversion. On the other hand, Kurzban and Houser [41] found more cooperative behavior for people high in neuroticism and low in conscientiousness. In a study by Lönnqvist, Verkasalo and Walkowitz [42] low neuroticism and high openness was associated with more cooperation in a prisoner’s dilemma game–but only in an incentivized variation of a game whereas personality had no predictive value for a hypothetical variation. The latter finding is especially interesting in relation to (potential) incentives for data sharing and their subjective importance for the individual researcher.

#### Dark Triad: Machiavellianism

Besides rather broad factors of personality, there also exist more specific conceptions of personality facets. In relation to problematic workplace behavior especially the so-called Dark Triad is often discussed [43]. The Dark Triad is defined by Machiavellianism (manipulative nature, cold selfishness, pure instrumentality), subclinical narcissism (grandiosity, vanity, superiority) and subclinical psychopathy (high impulsivity and thrill seeking along with low empathy), which are seen as constructs distinct from the Big Five [44]. Former findings suggest that Machiavellianism (in the following abbreviated as “Mach”) could be of special interest for the case of data sharing. Prior research on Mach showed its relation with unethical behavior [45] and persuasion [46]. In relation to general workplace behavior, high Mach was related to more counterproductive behavior [47], less organizational but more career commitment [48] and less willingness to share knowledge [49]. In that context, the concrete cases in which Mach matters were often explained by social dilemma and game theory [50–52]. Similarly, individuals with high Mach exhibited more selfish behavior and lower cooperation in social dilemmas [53]. Additionally, the findings of Bereczkei and Czibor [53] indicated, that high Mach individuals reacted more sensitively to situational cues and to the behavior of other people, which might explain their successful exploitation of others. Based on these findings, Mach could be problematic for data sharing, too. At present, data sharing is seen as good practice, but is only rarely practiced because sharing brings no personal career benefit (within an academic reputation economy). Thus, high Mach individuals with their selfish nature and high instrumental thinking should be more inclined to the reputation economy and less to the rather idealistic idea of making data openly available.

#### Social desirability

Another interesting personality facet in relation to data sharing is social desirability. Social desirability is the individual tendency of a person to give positive self-descriptions [54]. Like Paulhus [54] pointed out, we can differentiate between two sub-constructs of social desirability as a response style: self-deception versus impression management. Typically, self-deception relates to sexual and aggressive thoughts that are universal but often denied. On the other hand, impression management relates to social desirable behavior that is very evident and thus, normally not apt for self-deception. While self-deception shows relatively high associations with the Big Five, the correlations between the Big Five and impression management are weaker [54].

Social desirability (in the sense of impression management) is often integrated into self-reporting questionnaires as a control scale. In this context, social desirability describes the tendency of a person to answer in a way that will be viewed as “good behavior” while undesirable behavior will be under-reported. Besides its apparent value as a control variable in personality questionnaires, social desirability is also interesting for research on delicate topics like data sharing. Persons with high values in social desirability should be more inclined to give answers that create the impression of being a “good researcher” who shows “good behavior”.

#### Personality and sociodemographic variables

It is important to note that personality characteristics are associated with sociodemographic variables, namely age and gender. Even though personality factors are seen as rather stable characteristics, empirical research has found age-related differences and age-related changes [55,56]. Additionally, longitudinal studies [57] and cross sectional studies [55] suggested interactions between gender and age. There is also empirical evidence for gender-related differences in personality. For example, women reported higher neuroticism and agreeableness [58]. Additionally, gender might also directly influence the cooperation with data sharing. For example, several studies found that women showed more prosocial motives and were more cooperative than men [59]. However, there were also findings for stronger cooperative behavior in males [41]. A meta-analysis of gender and competitive behavior [60] indicated that gender-differences were reduced by situational factors. If the counterpart in negotiations applied a “tit-for-tat” bargaining strategy, women showed even more competitive behaviors than males. Based on these findings, we considered age and gender as basic control variables in our study on personality and data sharing.

### Aim of the study and research questions

Our research aim was to investigate the influence of the researcher's identity (in form of different personality domains) within the social dilemma of data sharing. As core factors of personality we investigated the influence of the so-called Big Five. Additionally, we included Machiavellianism and social desirability as personality facets. Based on prior findings on the influence of age and gender on personality, we also included these two sociodemographic variables in our analyses. As dependent variables we investigated four groups of indicators: the general attitudes toward data sharing, the willingness to share data, actual data sharing in the past and the importance of conditions of data sharing that might foster (enablers) or hinder (barriers) data sharing.

In the sense of the appropriateness framework, the researcher’s identity (in form of personality domains) should influence the view of the data sharing situation. We assessed this subjective perception in form of attitudes towards data sharing. The willingness to share data reflects the behavioral rules and heuristics the researcher wants to apply and thus is very close to the researcher’s identity. On the other hand, the “hard” behavioral indicator of whether the researcher actually shared his/her data in the past is not only dependent on the researcher’s identity and his/her subjective perception, but also on external institutional and systemic constraints. The fourth group of variables in the form of barriers (potential disadvantages) and enablers (potential incentives), gives insights how the individual researcher might be motivated to share his/her data in the future.

In the face of the incoherent findings on personality and social dilemmas, we formulated open research questions. Based on the mentioned four groups of dependent variables, the four research questions (RQs) were:

**RQ1 on attitudes towards data sharing:** How does personality influence attitudes towards data sharing?

**RQ2 on the willingness to share data:** How does personality influence the willingness to share data?

**RQ3 on actual data sharing in the past:** Hoe does personality influence actual data sharing in the past?

**RQ4 on the importance of enablers and barriers:** How does personality influence the individual importance of enablers and barriers for data sharing?

## Methodology

We addressed our research questions using a standardized questionnaire. In this section we describe the online survey, the participants, the assessment procedure, and the measurement of the variables.

All procedures performed in this study (involving human participants) were in accordance with the ethical standards of the institutional and/or national research committee and with the 1964 Helsinki declaration and its later amendments or comparable ethical standards. The German Institute for Economic Research (DIW) who hosted the survey and the data has no special ethic commission; the same is true for the other involved institutions (HIIG and ZBW). It is in the responsibility of the single researcher to respect the ethical standards of the Helsinki declaration. However, the survey was approved by the data protection officer of the DIW.

Informed consent was obtained from all participants included in the study by providing information about privacy issues on the first page of the online survey. After this privacy information the participants could simply skip the online survey if they disagreed and did not want to participate. No further written informed consent was recorded.

The assessed data were anonymous; no information about the participants’ names, addresses, IP-addresses or similar disclosures was recorded. Further assessed data that might provide any indication to the identity of the participants (e.g., in the open answers) will be removed before sharing the data for secondary data use.

### Description of the online survey and the assessment procedure

The questionnaire was newly designed. It was based on a previous study, consisting of a systematic review and a secondary data user survey [1]. The items in the questionnaire were mainly closed questions (multiple choice or rating scales). In addition we included some open questions where it was necessary or helpful. The first pre-version of the questionnaire was tested with a small sample of researchers to ensure its usability and the comprehensibility of the wording. After small modifications, a second round of pretesting was conducted with experts on data archiving and data reuse.

The questionnaire was answered in a self-administered manner. Participation was voluntary and there were no forced answers. The main part at the beginning of the survey was comprised of the questions on data sharing and related factors. This part of the questionnaire also included several questions that were not part of the study reported here (e.g., on secondary data use and publication behavior), but were assessed for other purposes.

The questions on data sharing included not only the personal attitudes towards data sharing but also under which conditions and with which groups the researches were willing (or not) to share and with which groups of people and under which conditions they had already shared their research data. A detailed description of the accordingly items follows in the subchapter on the dependent variables on data sharing. These questions were on a rather global level, i.e., we neither assessed the single interactions between researchers nor the individual consequences of data sharing. Rather the research questions examined the subjective views and personal decisions of researchers.

Sociodemographic variables and personality variables were assessed at the very end of the questionnaire. The assessment of personality variables was based on existing scales. In the face of the rather long questionnaire we chose short or shortened scales. A detailed description follows in the section on the assessment of personality variables. Please note that our sample was probably biased by the interest in the topic data sharing and the voluntary participation (without any reward). Thus, the *absolute* values of the personality measurements were probably not representative for the scientific community. However for the relative association (calculated by correlations and regression coefficients) between personality and the dependent variables on data sharing this systematic bias should not matter.

We formulated a German version and an analogous English version. The complete wording of the survey and a detailed description of all items can be found on the project’s Github-Page [61]. The online-survey was conducted from October to November 2014. It was administered via LimeSurvey. To recruit participants, we asked the faculty heads of 20 large, medium and small (with respect to the number of students) universities and universities of applied sciences to distribute the online survey among their researchers. We made the same request also to the scientific directors of the four biggest German research organizations, i.e., the Max Planck Society, the Leibniz Association, the Frauenhofer, and the Helmholz Association. Additionally, the link to the survey was uploaded on the website of the Science 2.0 research alliance (http://www.leibniz-science20.de/en/) and the German Data Forum (www.ratswd.de). The link was also sent to several mailing lists addressing researchers working with data. All answers were treated anonymously. We assured the respondents confidentiality in the invitation to the survey as well as at the beginning of the questionnaire.

### Participants

Overall, 2661 people started the questionnaire, but not all finished it. We excluded all respondents who failed to answer the questions on status, employer, and discipline or had less than 20% of the questions answered. We were left with 1564 valid cases (59% of all respondents).

**Fig 1:**
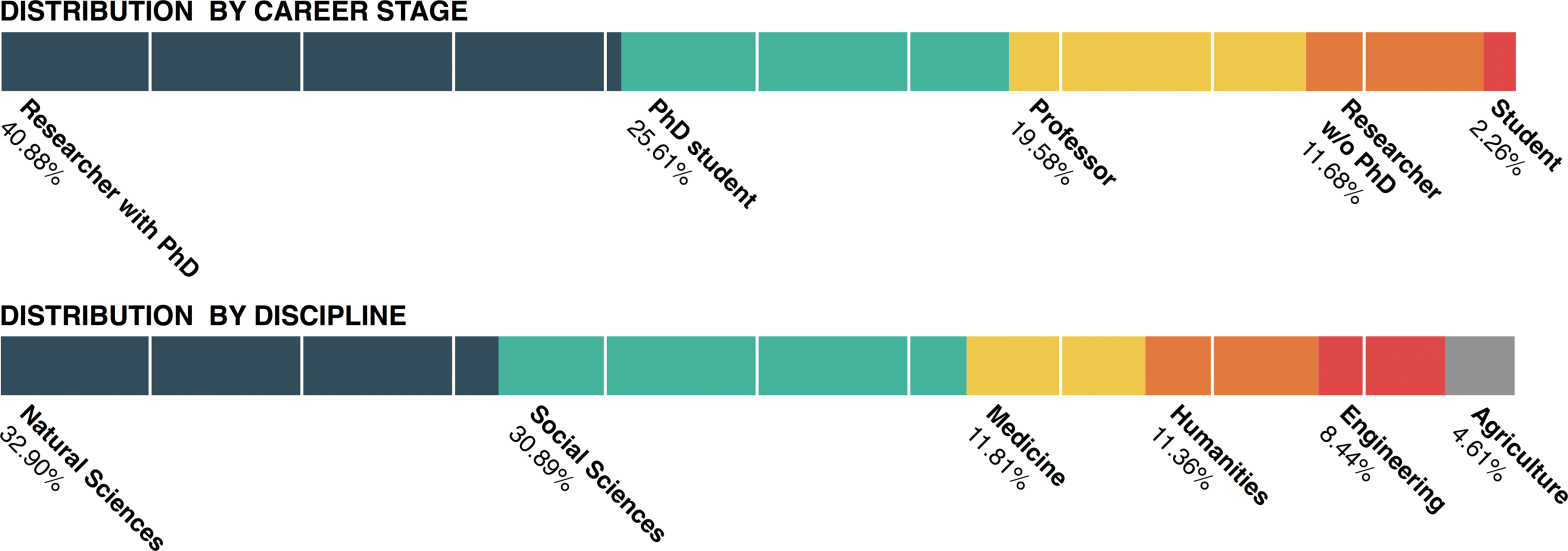
Sample composition. Sample composition by career stage and discipline.

Within the sample, 88% of the respondents were researchers from Germany and 12% were working in other countries. The relatively high number of researchers outside Germany can be explained by the recruitment via mailing lists and website postings. The sample contained slightly more males (56.78%) than females (43.22%). The average age of the participants was 38 years. Fig 1 shows the composition of our sample by academic status and discipline.

### Measurement of personality domains

To assess the personality variables we used already existing scales, partly in a modified manner. (For the exact wording of the items see the survey on the project’s Github-Page (61)).

The Big Five were assessed in a separate block after the general data sharing questions and the assessment of sociodemographic variables. Subsequent to the Big Five, we presented a separate section with the items on Mach and social desirability.

The *Big Five* were measured using a short scale designed by the German Socio-Economic Panel [62]. The sixteen items in the form of adjectives had to be rated on a 5-point Likert scale from 1 (does not describe me at all) to 5 (describes me perfectly). Additionally, the option “don’t know” was available.

The basis for the measurement of *Mach* was the scale developed by Dahling, Whitaker, and Levy [63]. For the German version we used the translation applied by Zettler and Solga [52]. However, in the face of our long survey on data sharing and the rather delicate wording of items we used only one item per subscale. The items took the form of statements. Each statement had to be rated on a 5-point Likert scale from 1 (strongly disagree) to 5 (strongly agree). Additionally, we provided a “don’t know” option.

*Social desirability* was measured by an existing short scale [64] with two items. The items assessed the desirable behavior towards colleagues, i.e., according to Paulhus [54], these items were related to social desirability in the sense of impression management (not self-deception). The items of the social desirability scale had the form of statements and were intermixed with the Mach items using the same answering format.

For each personality domain we calculated the mean score whereby high values indicate a high degree in the personality domain.

### Dependent variables on data sharing

The *attitudes* towards data sharing (see list below) included four aspects, two positive (A1 & A2) and two negative (A3 & A4). The items were formulated as statements that had to be rated on a five-point Likert scale from “1 (strongly disagree) to 5 (agree completely). Additionally, a “don’t know” option was provided.

The measured attitudes towards data sharing were:

- A1—researchers should share: “Researchers should generally publish their data.”
- A2—great contribution: “Freely available research data is a great contribution to scientific progress.”
- A3—more disadvantages: “I have more disadvantages than advantages when I share my data with others.”
- A4—deters from publishing: “It deters me from publishing when a journal requires the publication of my data.”

The participants’ *willingness* to share data was assessed with respect to six different target groups, namely researchers they personally know, researchers from their own institute/organization, researchers with a similar topic, all non-commercial researchers, commercial researchers, and the public. For each of the target groups, the participants had to indicate if they are willing to share their data with them or not.

Based on these detailed answers, we calculated two derived dichotomous variables: First, the “basic willingness”, i.e., the willingness to share with at least one of the target groups. If none of the target groups were indicated and the items before and after were answered, it was coded as unwilling to share. Second, the “extensive willingness”, i.e., the willingness to share with a broad audience in form of the public or/and all commercial researchers. If only the other smaller target groups or no target group were indicated and the items before and after were answered, it was coded as unwilling to share with a broad audience.

*Actual data sharing* behavior in the past was measured accordingly the same six target groups like willingness (see above). Analogous to the derived variables for willingness we calculated two derived dichotomous variables for data sharing: “basic data sharing” with at least one of the target groups and “extensive data sharing” with a broad audience (public or all non-commercial researchers).

The *enablers* of and *barriers* to data sharing were conceptualized as potential conditions, i.e., we asked if the participant would (enablers) or would not (barriers) share his/her data under specific conditions. The conditions (i.e., enablers and barriers) were formulated as sentences starting with “if” (see list below). The participants had to rate how appropriate the response was for them on a five-point Likert scale from 1 (does not apply at all) to 5 (applies completely). Additionally, the answering option “don’t know” was provided.

The assessed enablers (E) were:

- E1—known purpose of use: “if I knew what the data were going to be used for”
- E2—contact with researchers: “if sharing the data enabled me to get in contact with other researchers”
- E3—after publishing: “if I had enough time beforehand to publish on the basis of my data”)
- E4—known audience: “if I knew who would be able to access the data”
- E5—employer’s support: “if my employer supported me actively (e.g. by providing technical support, time)”
- E6—quotation: “if I were quoted in publications using my data”
- E7—co-authorship: “if I were given a co-authorship of articles using my data”
- E8—financial compensation: “if I received financial compensation for the effort”

The assessed barriers (B) were:

- B1—before publishing: “if other researchers could use my data to publish before me”
- B2—criticism / falsification: “if others could criticize or falsify my work”
- B3—misinterpretation: “if the data could be misinterpreted”
- B4—effort data collection: “if the data collection required considerable effort”
- B5—effort data sharing: “if a major effort was required to share data”

It’s important to note, that the enablers and barriers do not necessarily refer to already existing conditions; we asked for their *potential* relevance. Thus, the answers of the participants can be seen as indicating the importance of potential enablers and barriers for data sharing (independently of whether they already exist or not).

## Results

### Descriptive statistics and rational of the regression analysis

The research questions (RQ1 –RQ4) were addressed by multiple regression analysis.

As explained in subchapter 2.3 we used age and gender as sociodemographic variables as well as seven personality domains as predictors for the four groups of dependent variables. For the predictors and dependent variables with interval data, the means, standard deviations, and number of valid cases are provided in Table 1. For the dichotomous predictors and dependent variables, the number and the percentages of the categories as well as the total number of valid cases are presented in Table 2.

**Table 1:** Descriptive statistics for the interval-scaled predictors and dependent variables.

Variable (intervall)	m	s	n
Age	38.426	10.633	1309
Extraversion	3.425	.852	1225
Neuroticism	2.756	.856	1223
Openness	3.831	.651	1197
Conscientiousness	4.041	.654	1214
Agreeableness	3.922	.646	1215
Mach	2.271	.627	1183
Social desireability	3.226	.851	1196
A1: Researchers should share	4.101	1.005	1491
A2: Great contribution	4.340	.941	1449
A3: More disadvantages	2.318	1.158	1419
A4: Deters from publishing	1.939	1.145	1412
E1: Known purpose of use	3.269	1.381	1420
E2: Contact with researchers	3.038	1.237	1430
E3: After publishing	4.100	1.087	1420
E4: Known audience	3.182	1.387	1424
E5: Emloyer's support	3.585	1.247	1389
E6: Quotation	4.209	1.083	1420
E7: Co-authorship	2.862	1.417	1414
E8: Financial compensation	2.192	1.279	1393
B1: Before publishing	4.248	1.175	1409
B2: Critique/falsification	1.989	1.183	1402
B3: Misinterpretation	3.156	1.345	1383
B4: Effort data collection	2.568	1.264	1392
B5: Effort data sharing	3.549	1.191	1403

Descriptive statistics for the interval-scaled predictors and dependent variables: means (m), standard deviations (s), and number of valid cases (n).

**Table 2:** Descriptive statistics for the nominal-scaled predictors and dependent variables.

Variable (dichotom)	no (%)	yes (%)	n
Gender (male = no; female = yes)	750 (56.8%)	571 (43.2%)	1321
Basic willingness	4 (0.3%)	1404 (99.7%)	1408
Extensive willingness	442 (31.4%)	966 (68.6%)	1408
Basic data sharing	255 (18.2%)	1147 (81.8%)	1402
Extensive data sharing	1075 (68.8%)	327 (23.3%)	1402

Descriptive statistics for the nominal-scaled predictors and dependent variables: numbers (percentages in brackets) for yes versus no and total number of valid cases (n).

The values of the intercorrelations of the variables are listed in Table 3. We calculated Pearson correlations for interval data and Kendall-Tau correlations for nominal data. The intercorrelations showed that the Big Five, Mach, and social desirability were not independent of each other, and there were also significant associations with age and gender.

**Table 3:** Intercorrelations.

	1	2	3	4	5	6	7	8	9
1: Age	1	-.192**	-.015	-.111**	.136**	.026	-.018	-.080**	-.116**
2: Gender	-.192**	1	.149**	.089**	-.054*	.176**	.062*	.025	.017
3: Extraversion	-.015	.149**	1	-.234**	.219**	.108**	.092**	-.066*	-.017
4: Neuroticism	-.111**	.089**	-.234**	1	-.073*	-.166**	-.063*	.111**	.135**
5: Openness	.136**	-.054*	.219**	-.073*	1	.078**	.104**	-.001	-.103**
6: Conscientiousness	.026	.176**	.108**	-.166**	.078**	1	.173**	-.030	-.083**
7: Agreeableness	-.018	.062*	.092**	-.063*	.104**	.173**	1	-.179**	-.206**
8: Mach	-.080**	.025	-.066*	.111**	-.001	-.030	-.179**	1	.041
9: Social desirability	-.116**	.017	-.017	.135**	-.103**	-.083**	-.206**	.041	1
A1: Researchers should share	.055*	-.121**	.000	-.026	.082**	-.051	.0159	-.029	-.014
A2: Great contribution	.010	-.067*	.031	-.029	.059*	-.054	.010	-.030	-.003
A3: More disadvantages	-.017	.020	-.049	.065*	-.065*	-.006	-.052	.123**	.005
A4: Deters from publishing	-.036	.101**	.005	.070*	-.044	-.005	.036	.073*	-.052
Basic willingness	-.014	-.005	-.017	.040	-.046	-.046	-.036	-.027	.019
Extensive willingness	.007	-.136**	-.034	-.055*	.059*	-.093**	.003	-.050*	.050
Basic data sharing	.182**	-.144**	-.005	-.059*	.076**	-.009	.025	-.028	-.090**
Extensive data sharing	.138**	-.164**	-.032	-.014	.067**	-.048	-.049*	-.028	-.025
E1: Known purpose of use	-.002	.201**	.093**	.066*	-.013	.201**	.109**	.079**	-.111**
E2: Contact with researchers	-.036	.154**	.089**	-.004	.049	.149**	.066*	.186**	-.155**
E3: After publishing	-.074**	.138**	-.007	.029	-.035	.159**	.017	.092**	.031
E4: Known audience	.008	.188**	.153**	.032	.048	.182**	.081**	.081**	-.098**
E5: Emloyer's Support	-.099**	.131**	.065*	.010	.069*	.096**	.090**	.045	-.049
E6: Quotation	-.062*	.108**	.099**	-.017	.057*	.103**	.028	.046	.023
E7: Co-authorship	-.066*	.105**	.050	-.002	.079**	.099**	.088**	.130**	-.109**
E8: Financial compensation	-.147**	.130**	.085**	-.002	.025	.060*	.024	.163**	-.055
B1: Before publishing	-.042	.156**	-.002	.042	-.054	.162**	.060*	.067*	-.000
B2: Critique/falsification	-.148**	.191**	-.033	.094**	-.071*	.036	.037	.142**	-.120**
B3: Misinterpretation	-.064*	.128**	-.005	.052	-.018	.110**	.082**	.089**	-.138**
B4: Effort data collection	-.028	.085**	.017	.026	-.062*	.045	-.008	.153**	-.077**
B5: Effort data sharing	-.003	.052*	-.014	.025	.002	.016	-.001	.069*	.037

Intercorrelations between predictors and dependent variables: Pearson correlations for interval data and Kendall-Tau correlations for nominal data. We coded female with “1” and male “0”. Positive values mean that females’ scores are higher.

* p < .05

** p < .001

Since our data sample showed several significant associations between sociodemographic variables and personality domains, we included gender and age as a first block of predictors in the regression in order to control for the influence of sociodemographic variables. This procedure enables us to analyze the relatively “pure” influence of personality as a second block of predictors in the regression analysis. The following list gives an overview on the predictors of the regression analysis:

Block 1: sociodemographic variables

- age
- gender

Block 2: personality domains

- Extraversion
- Neuroticism
- Openness
- Conscientiousness
- Agreeableness
- Machiavellianism
- Social desirability

For the dependent variables answered by rating scales (attitudes; barriers and enablers) we calculated linear regression models. For the dichotomous dependent variables (willingness to share; actual data sharing in the past) we computed binary logistic regression models.

### RQ1 on attitudes towards data sharing

There was broad agreement with the statement (A2) that freely available research data is a great contribution to scientific progress–independent of sociodemographic variables and personality. This item was the one with the highest agreement levels (see Table 1 on descriptive statistics) and the non-significance of any predictor might be due to a ceiling effect.

However, we found significant differences for the other attitudes that relate to data sharing in a less abstract way: Females compared to males showed a lower agreement with the attitude (A1) that researchers should generally publish their data. In contrast, participants with a high score of openness show a higher agreement on this item. People with a high Mach-score, saw more disadvantages (A3) than advantages of data sharing. Additionally, people with a high Mach-score and females were more deterred from publishing (A4) if data sharing was required.

To sum up, we found a broad general acknowledgment of the scientific value of data sharing; however, the more concrete and practically relevant attitudes (related to the act of sharing, personal disadvantages and publishing) were influenced by the researcher’s gender and personality. The statistical values R^2^ of the regression models and the predictive values of sociodemographic variables and personality on the attitudes towards data sharing are listed in Table 4.

**Table 4:** Predictive values of sociodemographic variables and personality domains on the attitudes towards data sharing.

	A1: Researchers should share	A2: Great contribution	A3: More disadvantages	A4: Deters from publishing
	R^2^ = .025	R^2^ = .011	R^2^ = .021	R^2^ = .030
Gender	-.234**	-.080	-.006	.277**
	(.068)	(.062)	(.081)	(.081)
Age	.001	-.002	.000	.002
	(.003)	(.003)	(.004)	(.004)
Extraversion	.007	.024	-.015	.013
	(.039)	(.035)	(.046)	(.046)
Neuroticism	-.012	-.027	.077	.080
	(.039)	(.035)	(.047)	(.046)
Openness	.112*	.060	-.089	-.042
	(.049)	(.044)	(.059)	(.058)
Conscientiousness	-.060	-.075	.037	-.048
	(.049)	(.045)	(.059)	(.060)
Agreeableness	.000	-.018	-.063	.106
	(.050)	(.045)	(.060)	(.060)
Mach	-.009	-.051	.190*	.135*
	(.050)	(.046)	(.060)	(.060)
Social desireability	-.005	.001	-.045	-.066
	(.038)	(.034)	(.045)	(.045)

Predictive values of sociodemographic variables and personality domains on the attitudes towards data sharing: B (regression coefficient) and standard deviation of B in brackets. The values of the regression models (R^2^) are listed in the first row under the dependent variables.

* p < .05

** p < .001

### RQ2 on the willingness to share data

For the “basic willingness” to share data we found no significant effects of the predictors. However, this might be a statistical artifact (ceiling effect) since the vast majority of people were willing to share data with at least one of the listed target groups; only a very small group was basically unwilling to share (see descriptive statistics in Table 1).

By contrast, for the “extensive willingness” to share data with a broad audience, gender and personality mattered: Females were less willing. Similarly, people with high scores in neuroticism, conscientiousness and Mach reported a lower “extensive willingness". Conversely, openness and social desirability were positively related with the “extensive willingness”. The statistical values R^2^ of the regression models and the predictive value of sociodemographic variables and personality on the willingness to share data are listed in Table 5 on the left side.

**Table 5:** Predictive values of sociodemographics and personality domains on the willingness to share data and the actual data sharing.

	Basic willingness	Extensive willingness	Basic data sharing	Extensive data sharing
	R^2^ = .010	R^2^ = .051	R^2^ = .051	R^2^ = .047
Gender	.039	-.606**	-.466*	-.617**
	(1.655)	(.152)	(.178)	(.175)
Age	-.017	-.012	.042**	.022*
	(.071)	(.007)	(.010)	(.007)
Extraversion	.146	-.164	-.029	-.010
	(1.053)	(.089)	(.103)	(.097)
Neuroticism	1.582	-.193*	-.115	-.014
	(1.457)	(.087)	(.102)	(.097)
Openness	-2.006	.251*	.213	.219
	(1.847)	(.110)	(.127)	(.124)
Conscientiousness	-2.786	-.302*	-.048	-.107
	(2.626)	(.114)	(.132)	(.121)
Agreeableness	-.887	.103	-.021	-.277*
	(1.535)	(.112)	(.132)	(.123)
Mach	-1.128	-.229*	-.120	-.195
	(1.168)	(.113)	(.134)	(.126)
Social desireability	-.143	.189*	-.264*	-.041
	(.798)	(.085)	(.104)	(.094)

Predictive values of sociodemographics and personality domains on the willingness to share data and the actual data sharing: B and standard deviation of B (in brackets). The values of the regression models R^2^ (Cox & Snell) are listed in the first row under the dependent variables.

* p < .05

** p < .001

### RQ3 on actual data sharing in the past

Actual data sharing in the past was related with both sociodemographic variables: females (compared to males) reported about less “basic data sharing”and less “extensive data sharing”with a broad audience. Age showed a significant positive relationship with both dependent variables, i.e., older researchers reported about more “basic data sharing”and more “extensive data sharing“. For the personality domains we found a negative association between “basic data sharing”and social desirability, i.e., persons with high values in social desirability showed less “basic data sharing“. Additionally, participants with high scores in agreeableness showed less “extensive data sharing“. The statistical values R^2^ of the regression models and the predictive value of sociodemographic variables and personality for the actual data sharing in the past are listed in Table 5 (see above) on the right side.

### RQ4 on the importance of enablers and barriers

For enablers and barriers, both gender and age showed predictive value. For women all enablers and barriers were more important. Age was a significant positive predictor for the “known audience” condition, i.e., it was more important for older (compared to younger) persons to know the audience with access to the data. However, the employer’s support, financial compensation, and critique/falsification were less important for older people.

The predictive value of personality domains for the importance of enablers and barriers was partly consistent and partly inconsistent. Mach was the most prominent predictor for the importance of enablers and barriers: For people with a high Mach-score, nearly all enablers and barriers were more important (exception: no significant relationship between Mach and the enabler E6 “quotation”). For conscientiousness a similar but less pronounced pattern was found. Similarly, social desirability showed a significant influence on the importance of most enablers and barriers, however, in the form of a negative association: people with a high score in social desirability claimed that the conditions of data sharing were less important to them.

Extraversion, neuroticism, agreeableness and openness were only partly related to the conditions of sharing. It is worth notable that for the enablers”employers support” and “co-authorship” agreeableness was not the only significant (positive) predictor; openness and Mach were too and had the same direction of relationship.

The statistical values R^2^ of the regression models and the predictive value of sociodemographic variables and personality on enablers and barriers are listed in Table 6 (enablers) and Table 7 (barriers).

**Table 6:** Predictive values of sociodemographics and personality domains on enablers (E1-E8): B and standard deviation of B (in brackets). The values of the regression models (R^2^) are listed in the first row under the dependent variables.

	E1	E2	E3	E4	E5	E6	E7	E8
	R^2^ = .113	R^2^ = .111	R^2^ = .063	R^2^ = .103	R^2^ = .062	R^2^ = .034	R^2^ = .059	R^2^ = .071
Gender	.540**	.384**	.348**	.510**	.370**	.216*	.245*	.220*
	(.091)	(.081)	(.073)	(.091)	(.085)	(.073)	(.095)	(.086)
Age	.008	.001	.000	.010*	-.009*	-.002	-.005	-.014**
	(.004)	(.004)	(.003)	(.004)	(.004)	(.003)	(.004)	(.004)
Extraversion	.125*	.080	-.077	.191**	.005	.073	.040	.086
	(.052)	(.046)	(.042)	(.052)	(.048)	(.042)	(.054)	(.049)
Neuroticism	.157*	.010	.003	.129*	-.005	-.031	-.030	-.030
	(.052)	(.046)	(.042)	(.052)	(.048)	(.041)	(.054)	(.049)
Openness	-.078	.062	-.009	.019	.166*	.090	.171*	.067
	(.065)	(.058)	(.053)	(.066)	(.061)	(.052)	(.068)	(.062)
Conscientiousness	.274**	.177*	.216**	.230*	.091	.087	.100	.060
	(.066)	(.059)	(.053)	(.066)	(.062)	(.053)	(.069)	(.063)
Agreeableness	.181*	.099	.040	.143*	.169*	.044	.147*	.078
	(.067)	(.060)	(.054)	(.067)	(.062)	(.054)	(.070)	(.064)
Mach	.208*	.401**	.132*	.210*	.142*	.100	.325**	.363**
	(.067)	(.060)	(.054)	(.068)	(.063)	(.054)	(.071)	(.064)
Social desireability	-.157*	-.191**	.080	-.124*	-.024	.053	-.146*	-.075
	(.050)	(.045)	(.041)	(.051)	(.047)	(.040)	(.053)	(.048)

Predictive values of sociodemographics and personality domains on enablers (E1-E8): B and standard deviation of B (in brackets). The values of the regression models (R^2^) are listed in the first row under the dependent variables.

* p < .05

** p < .001

**Table 7:** Predictive values of sociodemographics and personality domains on barriers (B1-B5): B and standard deviation of B (in brackets). The values of the regression models (R^2^) are listed in the first row under the dependent variables.

	B1	B2	B3	B4	B5
	R^2^ = .063	R^2^ = .094	R^2^ = .068	R^2^ = .042	R^2^ = .016
Gender	.383**	.429**	.288*	.224*	.185*
	(.078)	(.078)	(.090)	(.085)	(.081)
Age	.003	-.010*	-.005	.002	.003
	(.004)	(.004)	(.004)	(.004)	(.004)
Extraversion	-.049	-.051	-.051	.046	-.022
	(.045)	(.045)	(.051)	(.048)	(.046)
Neuroticism	.019	.048	.117*	.036	-.004
	(.045)	(.045)	(.051)	(.049)	(.046)
Openness	-.077	-.063	-.027	-.111	.077
	(.056)	(.056)	(.065)	(.061)	(.058)
Conscientiousness	.228**	.031	.152*	.045	-.040
	(.057)	(.057)	(.066)	(.062)	(.059)
Agreeableness	.103	.051	.142*	.021	.031
	(.058)	(.058)	(.066)	(.062)	(.060)
Mach	.143*	.240**	.180*	.313**	.145*
	(.058)	(.058)	(.067)	(.063)	(.060)
Social desireability	.040	-.208**	-.238**	-.093*	.078
	(.044)	(.043)	(.050)	(.047)	(.045)

Predictive values of sociodemographics and personality domains on barriers (B1-B5): B and standard deviation of B (in brackets). The values of the regression models (R^2^) are listed in the first row under the dependent variables.

* p < .05

** p < .001

## Discussion

### Summary of the findings

Overall, sociodemographic variables as well as personality domains showed predictive value for the attitudes, the willingness to share, and the actual data sharing in the past as well as for the enablers and barriers. From the point of view of the appropriateness framework, our data demonstrated the influence of the researchers’ identity on how they perceive the situation measured by the attitudes towards data sharing. Interestingly, the positive value of data sharing was broadly accepted, regardless of personality or sociodemographic variables. But the more concrete attitudes with practical implications that address personal advantages and disadvantages were influenced by gender and personality. This pattern fits with the notion of data sharing as a social dilemma. The researchers acknowledge the general benefit, but the view of their personal role in this situation differed in relation to the researchers’ identity.

Similarly, the willingness to share data (which can be seen as the behavioral heuristic that the researcher wants to apply) and the actual data sharing behavior depended on the researchers’ personality and sociodemographic variables. However, the personality effects found here for willingness to share and actual data sharing were different from each other. This indicated that the actual data sharing was not directly dependent on the researchers’ subjective willingness but was also influenced by external factors. The most pronounced effects of personality (and sociodemographic variables) were found for the importance of enablers and barriers. On the one hand, this again underlines the influence of the researcher’s identity on the perception of the (data sharing) situation. On the other hand, the findings on potential enablers and barriers can provide the basis for a better understanding of possible incentives for the individual researcher.

It is important to note that there was not a consistent influence of personality, but rather very different patterns of effects for the individual indicators of the researcher’s identity. Additionally, some of the different personality domains showed partly an analogous pattern of effects. In light of the complexity of our findings, in the following we provide an interpretation of the overall pattern of effects for each of the predictors.

### Age and gender

The finding of more actual data sharing among older researchers was quite trivial, since older people normally have had (over their longer working lifetimes) more working experience and more opportunities to share their data. Similarly, it was not a surprise that financial compensation and employer's support was less important for older people since they normally have a higher position and salary. However, vice versa this also indicated that for younger researchers these could be essential criteria for data sharing and they need better funding opportunities for data management and archiving.

Overall, the findings for gender indicated that females (compared to males) were less open to data sharing: They showed more negative attitudes, a lower willingness, and less actual data sharing in the past. On the other hand, most enablers and barriers were more important for women. This pattern of findings implied that females’ general reservations towards data sharing might be connected with existing barriers and the need for more enablers. However, in the face of the ongoing debate on gender-roles and the status of women in male domains (like science) it seemed to be more appropriate to discuss possible interventions in a broader context. This discussion would be out of the scope of this paper (which focuses on personality) but appears to be a relevant future topic for gender research in the academic field.

### Extraversion and neuroticism

The two most popular personality domains, extraversion and neuroticism, had only minor predictive value for data sharing. Neither the general attitudes, nor actual data sharing in the past were related with extraversion or neuroticism.

For extraverted people there were only significant differences for the importance of two enablers: knowing the purpose of use and knowing the audience with access. Both enablers were related to the secondary data users, and their higher importance could be explained as relating to gregarious nature of extraverted people. That means, maybe these effects were less connected with data sharing but rather with the companionable thinking regarding the group of secondary data users.

The found effects for neuroticism (less willingness to share with a broad audience, higher importance of known audience, and fear of critique/falsification) could be explained with a higher sense of precariousness, which is one characteristic of neuroticism. Accordingly, a reasonable intervention for increasing the willingness to share data might be the provision of information on the concrete procedure of data sharing and secondary data usage. Additionally, the option of restricted access or specific use agreements could be integrated. Similarly, it might be helpful to give researchers a say or kind of veto option in relation to the secondary data use in order to lower their reservations and their worriedness about critique.

### Conscientiousness

While conscientiousness was not related to general attitudes towards data sharing and actual data sharing in the past, it was associated with a lower “extensive willingness”to share data with a broad audience and showed positive associations with some enablers and barriers. For conscientious people it was more important that they knew what the data were going to be used for and who had access to the data. In addition, they wanted to have enough time to publish their findings before data sharing and were deterred by the danger of misinterpretation. These findings could be well explained by the affinity for careful organization and planning of conscientious people. Similarly, the higher importance to get in contact with researchers probably had less to do with social interaction but rather might be seen as a possibility to open up new discussions and receive a kind of peer review. In light of this interpretation, highly conscientious people might be more willing to share their data with a broad audience if they can carefully plan their data sharing and if there is the option to receive additional feedback from peers. This would imply a close coordination with the secondary data users or a detailed negotiation of the concrete secondary data use. This could be either managed by offering personal contact with the secondary data users or by an appropriate data repository that offers the possibility of precise specifications on the conditions of secondary data use.

### Agreeableness

Agreeableness had no predictive value for attitudes towards data sharing or the willingness to share data. However, people with a high degree of agreeableness shared less often with the broad audience. Additionally, for agreeable people it was more important to know the purpose of use and the audience for data sharing. For agreeable people the employer's active support and co-authorship were more important, whereas they were more deterred from sharing if data could be misinterpreted. Overall this pattern of findings suggested high reservations towards data sharing. It is worth noting that agreeableness was the only personality domain that influenced actual data sharing with a broad audience.

At first sight these reservations towards data sharing were quite astonishing for agreeable people, who are normally defined by a trusting, helpful and generous nature. However, it might be the case that agreeable persons, with their helpful nature, more often had the experience that “just to be nice to colleagues” was somehow disappointing, for example by getting nothing in return even though the favor was quite demanding.

But another interpretation also is thinkable: it could be the case that agreeable persons were more inclined to comply with the standards of the given system, i.e., the reputation economy in which publications are of higher value than data sharing. (The similar findings on social desirability support this interpretation: see subchapter on social desirability below.) This interpretation would also explain why co-authorship and employers support were more important to them. Accordingly, researchers who share their data should receive formal credit.

### Openness

Openness was a positive predictor for the general attitude that researchers should generally share their data and for the willingness to share data with a broad audience. However, there were no significant effects for actual data sharing. Interestingly enough, openness only had predictive (positive) value for the enablers “employer’s support” and “co-authorship”. This pattern of findings suggested that the individual willingness is not enough, but rather the structural conditions also have to be improved. In this context, the higher importance of “employer’s support” might be one structural key to transform the higher willingness of open people into actual data sharing behavior. Similarly, the importance of co-authorship also could be interpreted in a system-related sense. As long as the currency of the academic system is publication output, co-authorship for shared data is the only possibility to make data sharing valuable.

### Machiavellianism

People with a high Mach score reported rather negative attitudes towards data sharing. Correspondingly, they were less willing to share with a broad audience. However, despite the lower willingness to share, Mach showed no influence on actual data sharing in the past. The latter finding was quite surprising. A look at the barriers and enablers revealed that nearly all of them were more important for persons with a high Mach score. On the one hand, the higher importance of enablers and barriers could be explained by the selfish and manipulative nature of high Mach individuals, i.e., in the case of data sharing it was important to high Mach persons to have as many advantages and as few disadvantages as possible to “profit” from sharing. On the other hand, the higher importance of enablers and barriers could also offer a possible explanation why high Mach people showed an equal data sharing rate despite their lower willingness: Maybe high Mach people had already made use of enablers and barriers to overcome their lower willingness to share data.

In this context a look at the findings on gender might be interesting. (Please note: in our sample gender and Mach were not correlated.) Analogous to high Mach people, women also showed a lower willingness to share and a higher importance of enablers and barriers. However, they reported less data sharing in the past. That means, high Mach individuals and females were both unwilling to share and indicated a high importance of the conditions of sharing, but this resulted in different actual behavior. While women simply refused to share data, for high Mach people the lower willingness did not cause less data sharing. One possible explanation might be that the lower willingness of females and high Mach people traced back to different subjective reasons. For example, for females the lower willingness might be connected with general working conditions (in science as a male domain) while the reasons of high Mach people are more related to their higher instrumentality. However, there is also another conceivable interpretation: It might be the case that high Mach persons were–more than women–able to make use of existing “resources” and to influence others to play by their rules. It seemed that high Mach individuals manipulated the conditions (barriers and enablers) in a way that compensated for their unwillingness because they received some personal benefit out of sharing. This interpretation also suggests that depending on their personality (e.g., their manipulative nature) researchers were more or less able and willing to make use of the existing academic system with its resources and possibilities.

### Social desirability

The effects on social desirability were quite surprising. On the one hand social desirability had no influence on any of the attitudes towards data sharing. However, social desirability was associated a higher willingness to share with a broad audience, but had no impact on the actual data sharing with a broad audience. Even more irritating, social desirability was associated with less “basic data sharing“. On first sight, this seemed counterintuitive since it suggested that less data sharing was the socially desirable case. In this relation, the results on enablers and barriers might give some insights: for people with high scores in social desirability, most enablers and barriers were less important. However, there was one notable exception: The enabler “after publishing” was more important for people with high scores on social desirability. This indicated that even though the willingness to share data might be seen as the “politically correct” opinion, it conflicted with another highly social desirable behavior in science, namely publishing. Maybe social desirability led to a higher focus on this maxim of the research community and thus data sharing was seen as an obstacle to optimizing publications. That means people with a high score in social desirability were neither for nor against data sharing, but neglected it because they were concentrated on publishing (as the higher social desirability behavior). Accordingly, the results on social desirability also suggested an interaction between the researcher’s personality and the academic system.

## Recommendations and outlook

The core question of this paper focused on the impact of the researcher’s personality on his/her data sharing behavior in the given academic reputation economy. Our results clearly demonstrated that data sharing was not only influenced by situational and organizational factors of the academic system (as reported in [1,2]), but also by the researchers’ age, gender, and personality.

Interestingly, we found different patterns of effects for the willingness to share data and the actual data sharing behavior. This indicated a complex interplay between the academic system and the individual researcher. Some personality factors influenced the willingness, but this did not necessarily cause a corresponding data sharing behavior. In these cases the conditions of sharing (i.e., barriers and enablers) could be seen as factors that counteract the higher willingness or compensate for the lower willingness.

In light of this interpretation we see the practical relevance of our findings for policy makers. If policy makers want to foster data sharing, it is not sufficient to concentrate only on global interventions. Rather they have also to consider the individual needs and apprehensions in relation to the researchers’ personality, for example:

- Interaction with data re-users could convince researchers that score high on consciousness to publish data, for instance in order to improve their own work. Interaction and feedback tools should therefore be included in online data repositories.
- Agreeable persons are more inclined to comply with the standards of a given system. Clear policy guidelines by funding agencies and scientific communities regarding data management and archiving would appeal to those researchers that stick to the implicit conventions of the scientific system, which currently is not to share data.
- Restricted access or clear use agreements would appeal to those researchers that score high on extraversion. This could be accomplished by using standard licenses and mandatory registration with data repositories.
- Employer's support, for instance through clear directives and additional services for data sharing, would support researchers that generally score high on openness.
- Finally, people with a high score in social desirability were neither for nor against data sharing, but neglected it because they focus on publishing results rather than "intermediate products". This clearly speaks for a general change towards giving more formal credit for socially desirable research practices, in this case data management and archiving.

Furthermore, the findings on Mach indicate that the change towards open science is not only about the establishment of new conditions but also about fairness and equal/democratic use of existing possibilities. That means, the existing conditions should be modified in a way that reduces the risk of free-riding and unintended use (e.g., by restricted access, use agreements) and makes data sharing a more social desirable behavior than publishing (e.g., by increasing the value of data citations). While our reported findings provide insights in the subjective perception of the conditions of data sharing, an important topic for future research is the interpersonal relation between the researchers, i.e., the relations and interaction between the researchers who share and the secondary data users. The work by Chen and colleagues [18] and by Wang and colleagues [19] showed that the assumed reputation of the interaction partner can foster cooperative behavior. According to the evolutionary game theory this in turn might result in the development of new collective strategies [15]. Applied to data sharing these findings suggest that the assumptions about the secondary data users and their reputation might influence the scientific cooperation in the sense of sharing the own research data. Thus, one decisional condition for data sharing might be if and to what degree the characteristics of the secondary data users are obvious.

Additionally, our findings also indicate that a holistic and effective policy should take the researcher’s personality into account and should appeal to the individual responsibility. The academic system should establish circumstances in which the researchers’ personal values and ethical standards are better aligned with the public benefits of data sharing. In the sense of the appropriateness framework, the researchers’ identity influences the individual decision about data sharing and open science in general. If the individual researchers have a stronger voice in data sharing, the future will show how much openness they really want.
